# Variability in Leaf Color Induced by Chlorophyll Deficiency: Transcriptional Changes in Bamboo Leaves

**DOI:** 10.3390/cimb46020097

**Published:** 2024-02-14

**Authors:** Peng-Kai Zhu, Mei-Yin Zeng, Yu-Han Lin, Yu Tang, Tian-You He, Yu-Shan Zheng, Ling-Yan Chen

**Affiliations:** 1 College of Architecture and Art, Fujian Agriculture and Forestry University, Fuzhou 350002, China18460376867@163.com (M.-Y.Z.); 52319026048@fafu.edu.cn (Y.-H.L.);; 2 College of Forestry, Fujian Agriculture and Forestry University, Fuzhou 350002, China

**Keywords:** chlorophyll deficiency, leaf color variation, *Bambusa multiplex*, transcriptome analysis, photosynthesis

## Abstract

The diversity of leaf characteristics, particularly leaf color, underscores a pivotal area of inquiry within plant science. The synthesis and functionality of chlorophyll, crucial for photosynthesis, largely dictate leaf coloration, with varying concentrations imparting different shades of green. Complex gene interactions regulate the synthesis and degradation of chlorophyll, and disruptions in these pathways can result in abnormal chlorophyll production, thereby affecting leaf pigmentation. This study focuses on *Bambusa multiplex* f. *silverstripe*, a natural variant distinguished by a spectrum of leaf colors, such as green, white, and green–white, attributed to genetic variations influencing gene expression. By examining the physiological and molecular mechanisms underlying chlorophyll anomalies and genetic factors in Silverstripe, this research sheds light on the intricate gene interactions and regulatory networks that contribute to leaf color diversity. The investigation includes the measurement of photosynthetic pigments and nutrient concentrations across different leaf color types, alongside transcriptomic analyses for identifying differentially expressed genes. The role of key genes in pathways such as ALA biosynthesis, chlorophyll synthesis, photosynthesis, and sugar metabolism is explored, offering critical insights for advancing research and plant breeding practices.

## 1. Introduction

The diversity of leaf characteristics, including leaf color, is indeed a key area of research in plant science [1,2]. The synthesis and functionality of chlorophyll are key factors determining leaf color [3]. During leaf development, the synthesis and degradation of chlorophyll is regulated by multiple genes [4]. In some plants, even though the normal chlorophyll synthesis genes may be present at the genetic level, abnormal gene expression regulation can lead to aberrant chlorophyll synthesis or functionality, ultimately affecting leaf color [5]. The synthesis and functionality of chlorophyll involve complex gene interactions and regulatory networks [6]. Disruptions in these interactions and networks by genetic factors can result in abnormal chlorophyll synthesis or functionality, consequently impacting plant leaf color [1,7,8].

In this study, we turn our attention to *Bambusa multiplex* f. *silverstripe* (hereafter referred to as Silverstripe), a naturally occurring variant of *Bambusa multiplex* that is prevalent in Southern China. Silverstripe is notable for its diverse leaf coloration, manifesting in shades of green, white, and a mix of green and white. This phenotypic diversity, underpinned by genetic variations, can lead to significant changes in gene expression through antagonistic interactions. Transcriptomic analyses offer a window into the underlying molecular mechanisms responsible for chlorophyll deficiencies and facilitate the identification of differentially expressed genes (DEGs) across various tissues [9]. As such, Silverstripe presents a unique model for investigating the genetic underpinnings of chlorophyll anomalies and related phenotypic expressions.

While gene expression’s influence on leaf color variation is well-documented across multiple species [10,11,12,13,14], systematic studies within the Bambusoideae subfamily, particularly concerning leaf color diversity, remain scarce. Moreover, within the Poaceae family, research has been predominantly focused on crops, with limited exploration of non-crop species [15,16,17,18]. Our research leverages transcriptome analysis, coupled with the quantification of photosynthetic pigments and nutrients, to unravel the physiological alterations and pinpoint DEGs associated with these chromatic variations. We anticipate that our findings will enrich the current understanding of the genetic determinants shaping leaf color diversity and chlorophyll content. This, in turn, could illuminate aspects of photosynthetic efficiency and overall plant vitality. Given the pivotal role of chlorophyll in photosynthesis, its scarcity can severely impair energy conversion and biomass accumulation [19,20,21,22]. Our exploration aims to contribute to a broader comprehension of plant growth, leaf morphology, and inform targeted breeding strategies, potentially leading to cultivars with enhanced photosynthetic efficiency and growth traits.

## 2. Methods

### 2.1. Plant Materials and Transcriptome Sequencing

The samples were collected from the Bamboo Botanical Garden of Fujian Agriculture and Forestry University, located in Cangshan District, Fuzhou City, Fujian Province, China (N26°05′, E119°14′). The plant experiments and field studies conducted in this study, including the collection of plant material, comply with relevant institutional, national, and international guidelines and legislation. We collected fresh leaves from five disease-free Silverstripe plants, each over four years old with stems exceeding 1.5 m in height. These plants were grown in full sunlight without any artificial light control. We selected representative leaves of green (G), white (W), and green–white (GW) for analysis, ensuring that the chosen leaves from each plant showed similar growth levels, despite variations in size. Before collecting the leaves, alcohol was uniformly sprayed on both sides of the leaves to ensure complete surface coverage. The alcohol was allowed to act on the leaf surfaces for approximately 30 s to 1 min for thorough disinfection. Subsequently, the alcohol-sprayed leaves were gently placed in deionized water to remove any residual alcohol and potential dead microorganisms from the surface, for a duration of about 2 min. The collected leaves were promptly mixed and then flash-frozen in liquid nitrogen. Subsequently, they were then stored at −80 °C. Total RNA from the leaves was extracted using the RNA prep Pure Plant Kit (Tiangen, Beijing, China). The quality of total RNA was assessed by 2% agarose gel electrophoresis. RNA concentration was measured using a NanoPhotometer^®^ spectrophotometer (IMPLEN, Westlake Village, CA, USA) and a Qubit^®^ RNA Assay Kit with a Qubit^®^ 2.0 fluorometer (Life Technologies, Carlsbad, CA, USA). RNA integrity was evaluated using an RNA Nano 6000 Assay Kit on an Agilent^®^ Bioanalyzer 2100 system (Agilent Technologies, Santa Clara, CA, USA), setting a benchmark RNA Integrity Number of 7 as the standard for quality assessment. Library construction was carried out using the NEB-Next^®^ Ultra^™^ RNA Library Prep Kit for Illumina^®^ (NEB, Ipswich, MA, USA). All samples were sequenced on the Illumina^®^ 6000 platform, generating 150 bp paired-end reads. RNA-seq for each type of leaf was performed with three technical replicates.

### 2.2. Measurement of Photosynthetic Pigments and Nutrient Concentrations 

We extracted photosynthetic pigments (chlorophyll a, chlorophyll b, chlorophyll (a + b), and carotenoids) from finely chopped fresh leaf tissues (0.25 g) using the acetone extraction method [23]. We measured the absorbance of the extracts at three different wavelengths (λ = 663 nm, λ = 645 nm, λ = 470 nm) using a UV-spectrophotometer (TU-1900, Persee, Beijing, China). Using the obtained optical density values, we calculated the concentrations of photosynthetic pigments (File S1).

For nutrient content assessment, we used dried samples (approximately 0.5 g of powdered tissue), with three biological replicates for each measurement. We determined the concentrations of total nitrogen (N) and sulfur (S) in the samples using an Elemental Analyzer (EMA502, VELP, Usmate Velate, Italy). We quantified the concentrations of other elements, including potassium (K), calcium (Ca), magnesium (Mg), copper (Cu), iron (Fe), manganese (Mn), zinc (Zn), and sodium (Na), using inductively coupled plasma mass spectrometry (ICP-MS) (Perkin NexION 300X, Waltham, MA, USA), as described (File S1).

### 2.3. Assembly and Function Annotation

We performed the initial processing of raw RNA-Seq reads using fastp v0.23.2 [24], removing sequences with adapters and sequences where more than 50% of the total length had bases with a Qphred ≤ 20, as well as sequences with an N-base percentage greater than 15%. The remaining clean reads were utilized for reference-based transcriptome assembly. De novo transcriptome assembly was carried out using Trinity v2.5.1 with default parameters [25]. Subsequently, Corset v1.07 [26] was employed for clustering to obtain non-redundant transcript groups. The longest transcript from each transcript group was chosen as a unigene. Custom Python scripts were utilized to rename each unigene for subsequent analyses. The completeness of the transcriptome was assessed using the Benchmarking Universal Single Copy Orthologs (BUSCO) tool v5.1.2 [27].

We annotated the transcripts using eggNOG v2.1.12 [28] based on diamond alignment (e-value ≤ 0.001) and InterProScan v5.63 with default parameters for functional annotation. We subjected transcripts that remained unannotated to a BLAST v2.10.0 search (e-value ≤ 1 × 10^−5^) [29] against the Arabidopsis thaliana (TAIR10) coding sequences (CDS) database. Additionally, we mapped the transcripts to the Kyoto Encyclopedia of Genes and Genomes (KEGG) pathway database using KofamKOALA [30] and the KAAS [31] server with default parameters.

### 2.4. Alignment, Quantification, and Identification of DEGs

In order to assess the utility of the reference transcriptome for gene expression quantification, we utilized the align_and_estimate_abundance.pl script from Trinity. This script utilizes bowtie2 v2.4.2 [32] for transcriptome alignment and RSEM v1.3.3 [33] for transcript abundance estimation. TBtools v2.012 [34] calculates the transcripts per kilobase million (TPM) normalized gene expression estimations, TPM values were used for the principal component analysis (PCA), while log2-transformed TPM values were used for gene expression graphs.

For statistical differential expression tests, count data were further analyzed using the R package DESeq2 v1.41.12 [35]; |log2 (Fold Change)| ≥1 and an FDR (false discovery rate) of <  0.01 were used as screening criteria. Subsequently, common DEGs among different types were identified using the Venn module within TBtools v2.012.

### 2.5. Expression Clustering and Functional Enrichment

We utilized TPM values for the clustering of common DEGs and performed the analysis using Mfuzz v2.60.0. We conducted Gene Ontology (GO) and Kyoto Encyclopedia of Genes and Genomes (KEGG) enrichment analysis using ClusterProfile v4.10.0 [36]. To create gene expression graphs, we employed the heatmap module within TBtools v2.012.

## 3. Results

### 3.1. Photosynthetic Pigments and Nutrient Concentrations of Three Color Types

**Figure 1:**
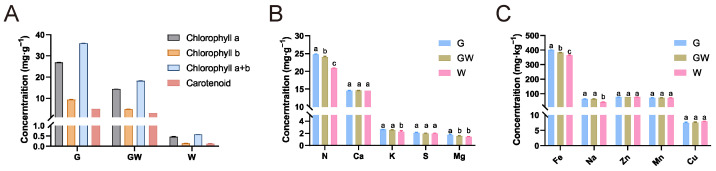
Photosynthetic pigments and nutrient levels. (**A**) Concentrations of photosynthetic pigments. (**B**) Concentrations of micronutrient. (**C**) Concentrations of macronutrient. Different lowercase letters above the bars within each content denote statistically significant differences, as determined by ANOVA (*p* < 0.05).

To better comprehend the underlying mechanisms and physiological responses associated with the chlorophyll-deficient phenotype, we carried out detailed quantitative assessments of photosynthetic pigments and nutrient levels in leaves exhibiting three different color variations. The analysis revealed a decreasing trend in the content of photosynthetic pigments in G, GW, and W types, consistent with their lack of green pigmentation (Figure 1A). There were no significant differences in the content of Ca, S, Zn, Mn, and Cu among the three types. However, significant differences were observed in the content of N and Fe among the types, with a decrease corresponding to the degree of chlorophyll deficiency (Figure 1B,C). N is a key element in the amino acids and proteins that constitute chlorophyll molecules, while Fe is an essential trace element in the process of chlorophyll synthesis [37,38]. Therefore, the observed variations might be due to an impediment in the transport and metabolism of these elements in tissues lacking chlorophyll, indirectly affecting the synthesis of chlorophyll and photosynthesis. Additionally, the content of Mg in GW and W types also decreased (Figure 1C). Since Mg is a core component of the chlorophyll molecule, this observation might be a consequence of the reduced chlorophyll content in the tissues. Concurrently, the decrease in K and Na content in W type (Figure 1C) may suggest changes in broader metabolic pathways and ion transport mechanisms potentially caused by chlorophyll deficiency and obstructed photosynthetic pathways.

### 3.2. Summary of Sequencing Data and Assembly

After trimming and quality assessment of the raw reads, we obtained a total of 76.17 GB (gigabytes) of high-quality reads (Table S1). The assembly revealed 336,879 unique unigenes, which were subsequently labeled Bm000001 to Bm336879. Analysis of these unigenes showed that 29.97% were between 201 and 500 bp, 33.08% were between 501 and 1000 bp, 22.89% were between 1001 and 2000 bp, and 14.06% exceeded 2000 bp in length, as indicated in (Table S2). The assembly’s N50 value was determined to be 1586 bp, with a BUSCO score of 89.0% (Figure S1).

### 3.3. Expression Quantification and Classification

**Figure 2:**
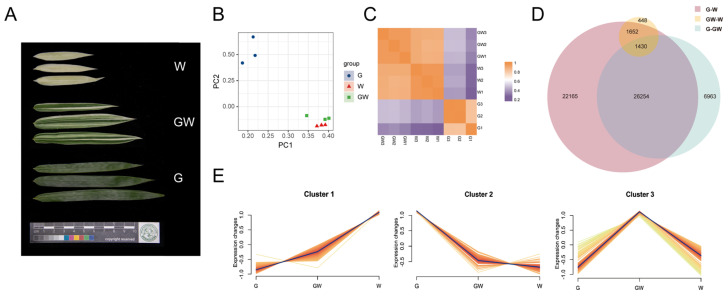
(**A**) Three leaf color types. (**B**) Principal component analysis (PCA) distribution of three color types. (**C**) Correlation heatmap of three color types. (**D**) Venn plot illustrating the filtration of common differentially expressed genes (DEGs) among different combinations. (**E**) A cluster analysis of transcripts per kilobase million (TPM) values for the common DEGs. G: green leaves. GW: green–white leaves. W: white leaves.

To elucidate the transcriptional mechanisms underlying the heterogeneity of leaf color in bamboo, we conducted an RNA-seq experiment covering three leaf colors (Figure 2A). By aligning and quantifying the sequencing data, we obtained count data and calculated TPM values (Tables S3 and S4). To further investigate the differences between the chlorophyll-deficient type and the normal type, we performed PCA analysis on the expression data of the nine transcriptome samples and generated a correlation heatmap to study their variation and similarity. In the scatterplot between principal component (PC) 1 and PC 2, it is evident that the three replicates of each leaf color type cluster closely together, indicating similarities in gene expression levels. Additionally, there is a notable distinction between G and GW or W, whereas the GW and W exhibit closer distribution and smaller differences (Figure 2B).

Furthermore, the correlation heatmap demonstrates high within-group correlation for samples of the same leaf color type, while the inter-group correlation is comparatively lower (Figure 2C). In line with the PCA analysis results, the correlation between W and GW is significantly higher than the correlation between G and either of them.

### 3.4. Identification of DEGs

Significant differences in transcript abundance were identified by DESeq2 based on the count data (Figure S2). Among the different combinations, the G vs. W combination had the highest number of DEGs. There were a total of 22,523 upregulated genes and 28,978 downregulated genes in this combination. In contrast, the GW vs. W combination had the lowest number of DEGs, with 2846 genes upregulated and 1326 genes downregulated. Additionally, in the G vs. GW combination, there were 12,220 upregulated genes and 23,069 downregulated genes (Figure S1). These results suggest that as the differences in leaf color become more pronounced in Silverstripe, the number of DEGs increases.

Further analysis revealed that there were 1430 common DEGs shared among the three combinations (Figure 2D). Moreover, cluster analysis showed that these DEGs could be categorized into three clusters (Figure 2E). Furthermore, there were 405 genes and 609 genes that exhibited the highest expression levels in the W and G groups, respectively, and their expression patterns consistently upregulated or downregulated with the degree of leaf whitening (Figure 2E and Table S5). This implies that these genes may be associated with the proportion of leaf white area and are considered key genes related to chlorophyll deficiency.

### 3.5. Function Annotation and Enrichment

To gain a deeper understanding of the molecular mechanisms behind physiological changes and to explore the biological significance of key genes in more detail, we initiated functional annotation for all unigenes. A total of 170,574 unigenes were successfully associated with GO terms (Table S6), accounting for 50.63% of all unigenes. However, only 53,046 unigenes, representing 15.75% of the total unigenes, could be effectively annotated to corresponding KEGG pathways. These pathways were distributed across 121 different categories (Table S7).

**Figure 3:**
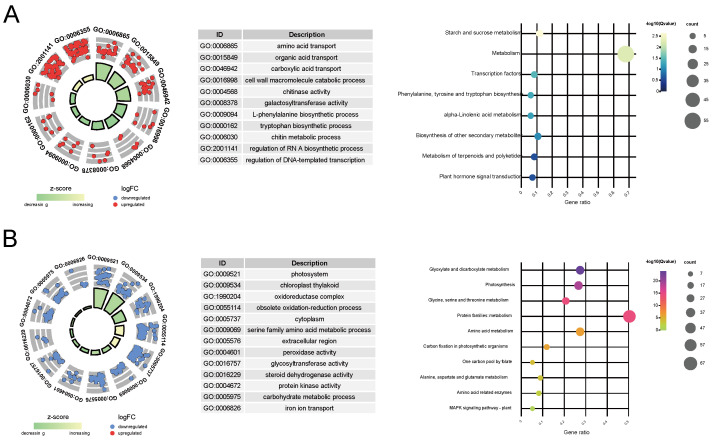
Functional enrichment analysis of key genes identified in the study. (**A**) Gene Ontology (GO) and Kyoto Encyclopedia of Genes and Genomes (KEGG) pathway enrichment for genes that are upregulated. (**B**) GO and KEGG pathway enrichment for genes that are downregulated. The left panels display the results of the GO enrichment analysis, while the right panels show the KEGG enrichment analysis outcomes. In the left panels, the density of the points correlates with the quantity of genes linked to each specific functional category. The radial distance from the center correlates with the extent of gene differential expression across phenotypes. For upregulated genes, a smaller radial distance signifies a higher degree of differential expression, whereas for downregulated genes, a larger radial distance indicates a more pronounced expression difference.

To further explore the intricate relationship between key genes and the variation in leaf color, we conducted KEGG pathway and GO enrichment analyses. In W, the relative upregulated key genes were subjected to GO functional enrichment, revealing their enrichment in 145 GO terms, which could be categorized into five main classes: amino acid and organic acid transport (e.g., amino acid transport, amino acid transmembrane transport), cell wall metabolism (e.g., cell wall macromolecule catabolic process, cell wall macromolecule metabolic process, chitinase activity), wall-related enzyme activity (e.g., chitinase activity and galactosyltransferase activity), metabolic pathways (L-phenylalanine biosynthetic process, chitin metabolic process), and the regulation of biological processes (e.g., RNA-directed RNA polymerase complex, regulation of DNA-templated transcription) (Figure 3A and Table S8). Additionally, KEGG analysis only enriched eight pathways. This might be due to the relatively smaller number of unigenes included in the KEGG annotation background. However, similar to the GO enrichment results, these pathways can be simplified into categories such as transcription factor (TF) regulatory pathways (e.g., transcription factors and plant hormone signal transduction), organic acid metabolism (e.g., phenylalanine, tyrosine, and tryptophan biosynthesis and alpha-linolenic acid metabolism), and starch and sucrose metabolism (Figure 3A and Table S8).

Among the downregulated key genes, GO functional enrichment analysis revealed their enrichment in 175 GO terms, primarily falling into four categories: photosynthesis-related pathways (e.g., photosystem, photosynthetic membrane), chloroplast-related processes (e.g., chloroplast thylakoid, plastid stroma), redox reactions (e.g., oxidoreductase complex, photosystem II oxygen evolving complex, oxidoreductase activity), and metabolic processes (e.g., glycine catabolic process, serine family amino acid catabolic process) (Figure 3B and Table S9). Correspondingly, KEGG pathway analysis enriched 17 pathways, largely related to photosynthesis (e.g., photosynthesis–antenna proteins, photosynthesis proteins), amino acid metabolism (e.g., alanine, aspartate, and glutamate metabolism, arginine biosynthesis), and carbon and energy metabolism (e.g., glyoxylate and dicarboxylate metabolism, one carbon pool by folate) (Figure 3B and Table S9). 

The result of GO and KEGG functional enrichment indicates that as chlorophyll content decreases in the leaves, genes involved in TFs’ regulatory and organic acid metabolism pathways are significantly upregulated, while genes related to photosynthesis and carbohydrate metabolism pathways may be downregulated as the extent of chlorophyll deficiency increases.

## 4. Discussion

In studies of variegated plants like Silverstripe, leaf color variations are largely due to differences in pigment types, amounts, and distribution [14]. Unlike typical pigment-driven color changes, Silverstripe’s white leaves are notably caused by chlorophyll deficiency, offering unique insights into the mechanisms of leaf color variation. Analysis of differentially expressed genes (DEGs) across its color variants revealed a predominance of downregulated genes in chlorophyll-deficient phenotypes. This suggests that chlorophyll deficiency not only affects photosynthesis but also influences gene expression, potentially disrupting key biological processes. Functional enrichment analysis indicated that upregulated genes in chlorophyll-deficient leaves are linked to transcriptional regulation and organic acid metabolism (Figure 3A), highlighting adaptive metabolic adjustments in response to reduced chlorophyll synthesis. This connection between organic acid metabolism and chlorophyll synthesis, particularly through the precursor 5-aminolevulinic acid (ALA), underscores the complex interplay between metabolic pathways and pigment synthesis in leaf color determination [39,40,41].

In the following discussion, we will explore the ALA biosynthesis pathway, the transcriptional changes related to chlorophyll deficiency, and their impact on photosynthesis and sugar metabolism.

### 4.1. Chlorophyll Biosynthesis in Chlorophyll Deficiency in Bamboo Leaves

**Figure 4:**
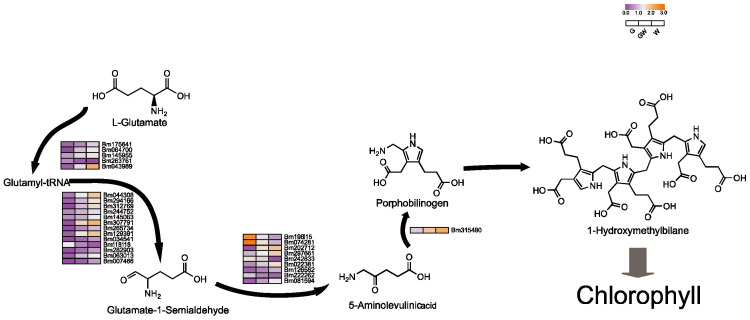
Key genes involved in the tetrapyrrole biosynthesis pathway. Black arrows indicate substance conversion. Gray arrows indicate substance conversion with omitted intermediate products.

Pyrrole, essential for synthesizing chlorophyll and hemoglobin, involves a complex metabolic pathway from ALA [42,43]. Surprisingly, our findings reveal increased expression of several structural genes within this pathway in chlorophyll-deficient tissues, particularly in the W phenotype (Figure 4). This upregulation might indicate a compensatory mechanism to maintain chlorophyll precursors and chloroplast functionality despite chlorophyll deficiency. This suggests a potential role for these genes, either in inhibiting enzyme synthesis within the pathway or in hastening the breakdown of chlorophyll precursors (Table S10). Furthermore, to offset photosynthesis loss due to chloroplast impairment, plants might boost the production of alternative pigments. Notably, the pyrrole pathway yields critical molecules like hemoglobin, vitamin B12, and carotenoids, which are vital for respiration, metabolism, antioxidant defense, and photoprotection in plants [42,44]. The increased expression of these genes could thus help preserve chlorophyll precursor availability, ensuring chloroplast functionality and plant stability. Interestingly, a subset of genes in the ALA pathway showed higher expression in G phenotypes (Figure 4), hinting at a complex interaction where chlorophyll scarcity might suppress certain light-dependent genes, potentially affecting ALA synthesis gene regulation [45].

Additionally, N is essential for enzyme synthesis in chlorophyll production [46,47]. Chlorophyll’s structure, comprising four N-bound pyrrole rings around a Mg atom, underscores Mg’s role in its synthesis and stability [48,49]. Fe serves as a cofactor in early chlorophyll biosynthesis stages, particularly in the conversion of protochlorophyllide to chlorophyll by the enzyme divinyl protochlorophyllide reductase (DPOR) [23,50]. Thus, reductions in N, Mg, and Fe could hinder chlorophyll synthesis, potentially due to genetic variations or as an adaptive response to chlorophyll deficiency, reflecting its direct impact on plant physiology and metabolism.

### 4.2. Chlorophyll Deficiency Induces Alterations in the Photosynthetic Process

**Figure 5:**
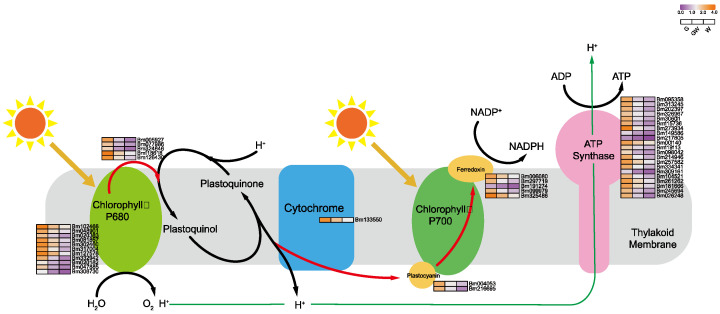
Key genes involved in photoreaction process in photosynthesis. Black arrows indicate substance conversion. Red arrows indicate electron transfer. Green arrows indicate H^+^ transfer.

**Figure 6:**
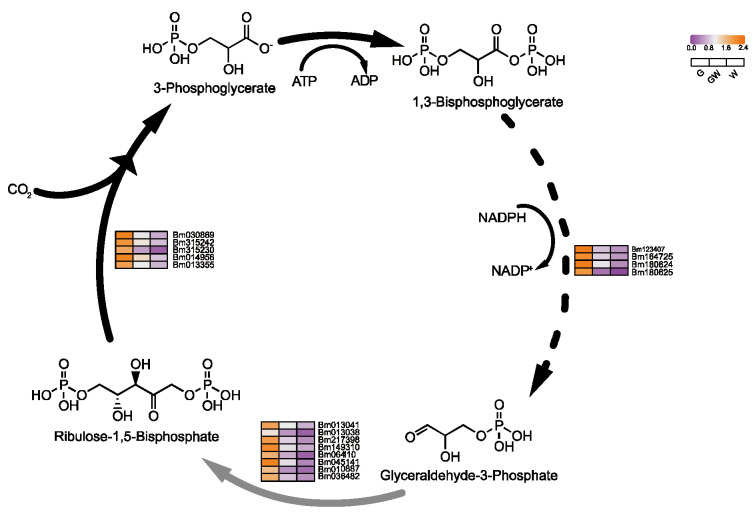
Key genes involved in the Calvin cycle process in photosynthesis. Black arrows indicate substance conversion. Gray arrows indicate substance conversion with omitted intermediate products. The dashed lines in the figure represent the departure of some metabolites from the cycle.

The lack of chlorophyll in white leaves leads to downregulated DEGs, impacting the light-dependent reactions of photosynthesis and carbon metabolism [51]. Predominantly downregulated genes include those involved in photosystem I and the Calvin cycle’s reduction phase (Figure 5 and Figure 6), such as NADP+ reductase and glyceraldehyde-3-phosphate (G3P) dehydrogenase, essential for energy conversion and carbon fixation. This downregulation suggests a decrease in photosynthetic efficiency and carbohydrate production in white leaves, aligning with known impacts of chlorophyll deficiency [52,53]. Furthermore, reduced Fe content in chlorophyll-deficient leaves may impair chlorophyll biosynthesis and the photosynthetic electron transport chain, as Fe is critical in iron–sulfur proteins for electron transfer [54,55]. These findings highlight the interconnected effects of chlorophyll deficiency on photosynthesis and plant metabolism.

The observed downregulation of photosynthesis-related genes in chlorophyll-deficient leaves indicates an adaptive inhibitory response, likely mediated by complex signaling pathways. This includes light signal transduction and hormonal regulation, which are crucial for adjusting to reduced photosynthetic activity [47,48,49]. Such differential gene expression in white leaves underscores a sophisticated regulatory mechanism aiming to plant energy balance and support growth under chlorophyll-limited conditions. This insight opens avenues for further exploration of the intricate network that orchestrates photosynthesis regulation, highlighting the resilience of plants in adapting to environmental stresses.

### 4.3. Differential Glycolysis in Chlorophyll-Deficient Phenotypes

**Figure 7:**
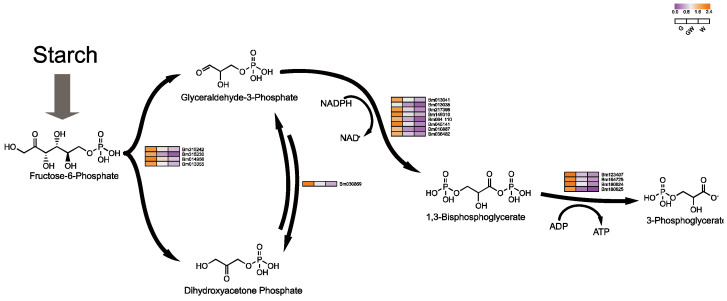
Key genes involved in plant glycolysis process. Black arrows indicate substance conversion. Gray arrows indicate substance conversion with omitted intermediate products.

An intriguing aspect of our study is the notable downregulation of genes crucial for glycolysis in white leaves, suggesting an adaptive shift in metabolic pathways to sustain energy production despite compromised photosynthesis. This adjustment aligns with the understanding that plants modulate sugar metabolism to maintain energy homeostasis under photosynthetic stress [56]. The glycolytic pathway, essential for energy provision and metabolic processes [57], intertwines with the Pentose Phosphate Pathway, which shares intermediates with the Calvin cycle (Figure 7), facilitating flexible interconversion to meet cellular demands [58,59].

Conversely, the observed downregulation of sugar metabolism-related genes in white leaves might stem from the need to mitigate oxidative stress, which can intensify in the absence of chlorophyll, leading to cellular damage [6]. This response likely aims to reduce reactive oxygen species production by curtailing sugar metabolism, as evidenced by the downregulation of key glycolytic enzymes like phosphoglycerate kinase and mutase (Table S11). Moreover, this metabolic regulation might also influence leaf senescence, as altered sugar levels can trigger aging processes in chloroplast-deficient tissues [56,60], suggesting a complex interplay between metabolic adjustments and plant stress responses.

## 5. Conclusions

Leaf color diversity, particularly in *Bambusa multiplex* f. *silverstripe* with its green, white, and green–white leaves, is primarily governed by chlorophyll synthesis and its regulatory genes. Our RNA-seq analysis revealed that the white phenotype has diminished photosynthetic and sugar metabolism activities, suggesting a link to chlorophyll synthesis disruption. These variations imply genetic influences on chlorophyll-related gene expression, affecting plant vitality and adaptation.

Current research underscores the need for a deeper understanding of the transcriptional control mechanisms associated with chlorophyll deficiency. Future research endeavors should extend to metabolomics and proteomics to elucidate the post-transcriptional modifications and the wider metabolic consequences of chlorophyll deficiency. This comprehensive approach will provide a fuller picture of the physiological adaptations in bamboo color variants. Furthermore, the observed leaf color variations in Silverstripe may also be influenced by environmental factors. Conducting population genetic analyses could be instrumental in unraveling the complex interplay between phenotypic changes and environmental influences, enriching our knowledge of the genetic and metabolic networks shaping leaf coloration and overall plant development.

## Data Availability

The datasets generated or analyzed during the current study are available in the NGDC (National Genomics Data Center) repository, PRJCA023136. Further information may be requested from the corresponding author.

## Supplementary Materials

The following supporting information can be downloaded at: https://www.mdpi.com/article/10.3390/cimb46020097/s1.
