# Supplementary material for: Variability in Leaf Color Induced by Chlorophyll Deficiency: Transcriptional Changes in Bamboo Leaves
Source: Curr Issues Mol Biol. 2024 Feb 14;46(2):1503–15. doi: 10.3390/cimb46020097 (PMC10888276; doi:10.3390/cimb46020097)
Supplement: Supplementary file 1 [file cimb-46-00097-s001.zip › File S1.pdf]

Detailed Measuring Methods

Measurement of photosynthetic pigment content

For the experiment, 0.25 grams of fresh leaves from three different color varieties were gathered to form a single sample. These leaves were then meticulously chopped into small pieces, each measuring 0.1 × 0.1 cm. To ensure accuracy, this process was replicated three times for each variety. Following this, each sample was submerged in a 25 mL mixture of acetone and anhydrous ethanol, maintaining a 1:1 volume ratio. The samples were then left undisturbed in a dark environment for 48 hours, allowing the leaf pieces to fully bleach until they turned completely white.

The next step involved determining the chlorophyll content of these samples. This was achieved by measuring the optical density (OD) of the extracted liquid at three different wavelengths: 663 nm, 645 nm, and 470 nm, using a UV-spectrophotometer. For each sample, the OD was measured thrice, culminating in a total of 36 readings. The overall chlorophyll content was then calculated based on specific equations derived from these readings.

$$Chl_a = (12.72 \times A_{663} - 2.59 \times A_{645}) \times \mathrm{V} / (1000\ \mathrm{W}) \quad (S1)$$

$$Chl_b = (22.88 \times A_{645} - 4.67 \times A_{663}) \times \mathrm{V} / (1000\ \mathrm{W}) \quad (S2)$$

$$Chl_{a+b} = (20.29 \times A_{645} + 8.04 \times A_{663}) \times \mathrm{V} / (1000\ \mathrm{W}) \quad (S3)$$

$$Cars = (A_{470} \times \mathrm{V} / \mathrm{W} - 2.05 \times Chla - 114.8 \times Chlb) / 245 \quad (S4)$$

Here, Chl_a, Chl_b, Chl_{a+b}, and *Cars* represent chlorophyll a, chlorophyll b, total chlorophyll content, and carotenoid content ($mg \cdot g^{-1}$), respectively. V stands for the total volume of liquid extract (mL), and W stands for the weight of one sample (g).

Measurement of nutrients

Leaves from each phenotype were collected and subjected to a 48-hour oven drying process at 75 °C to achieve a constant weight. The dried samples were then ground into a fine powder to pass through a 100-mesh sieve. A 20 mg portion of this powdered sample was used for the determination of total nitrogen (N) and sulfur (S) content using an Elemental Analyzer (EMA502,

VELP, Italy). For the analysis of other minerals, 0.5 g of the powdered sample was placed into a 40 mL crucible and ashed at 500 °C for 16 hours. The ashed samples were cooled to a temperature below 200 °C and then equilibrated with 15 mL of 0.5 M HCl at room temperature for 30 minutes, utilizing an adjustable macro-pipette to digest the ashes. The resultant sample solution was decanted into 25 mL glass tubes and stored at a temperature below 4 °C until analysis. This solution was analyzed for potassium (K), calcium (Ca), magnesium (Mg), copper (Cu), iron (Fe), manganese (Mn), zinc (Zn), and sodium (Na) using Inductively Coupled Plasma Mass Spectrometry (ICP-MS) (Perkin NexION 300X, Waltham, MA, USA). An external calibration was conducted using standard solutions within a range of 0–500 $\mu g \cdot L^{-1}$. To ensure the accuracy of our measurements, both these standard solutions and a quality control standard (Control Standard 4, SCP Science) were employed. This approach consistently yielded recoveries ranging from 90% to 110%.
