# Supplementary material for: Variability in Leaf Color Induced by Chlorophyll Deficiency: Transcriptional Changes in Bamboo Leaves
Source: Curr Issues Mol Biol. 2024 Feb 14;46(2):1503–15. doi: 10.3390/cimb46020097 (PMC10888276; doi:10.3390/cimb46020097)
Supplement: Supplementary file 1 [file cimb-46-00097-s001.zip › Supplementary Figures S1 and S2.pdf]

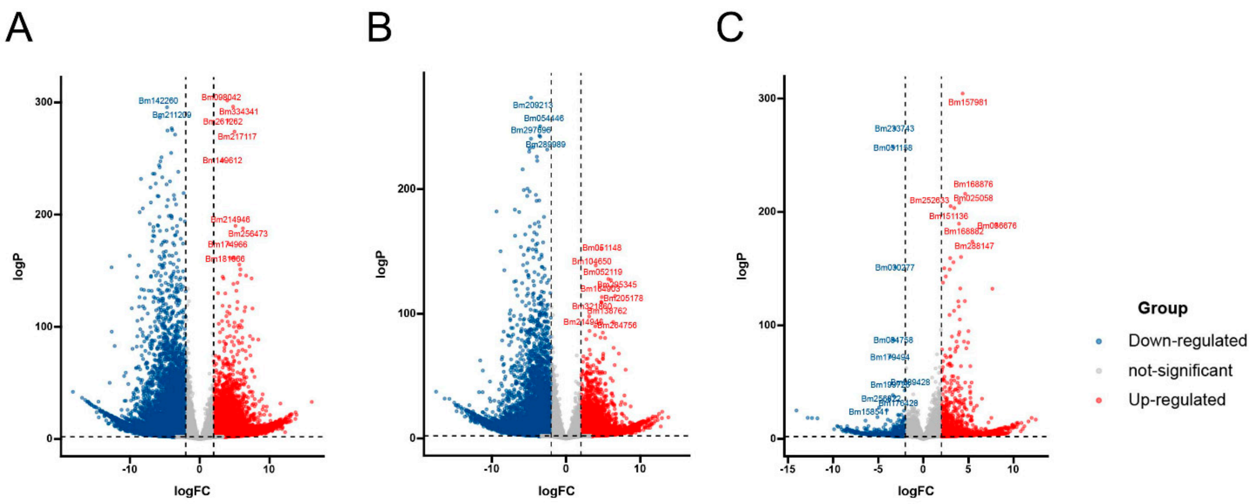

Figure S1. (**A–C**) Benchmarking Universal Single-Copy Orthologs (BUSCO) assessment results of transcriptome assembly.

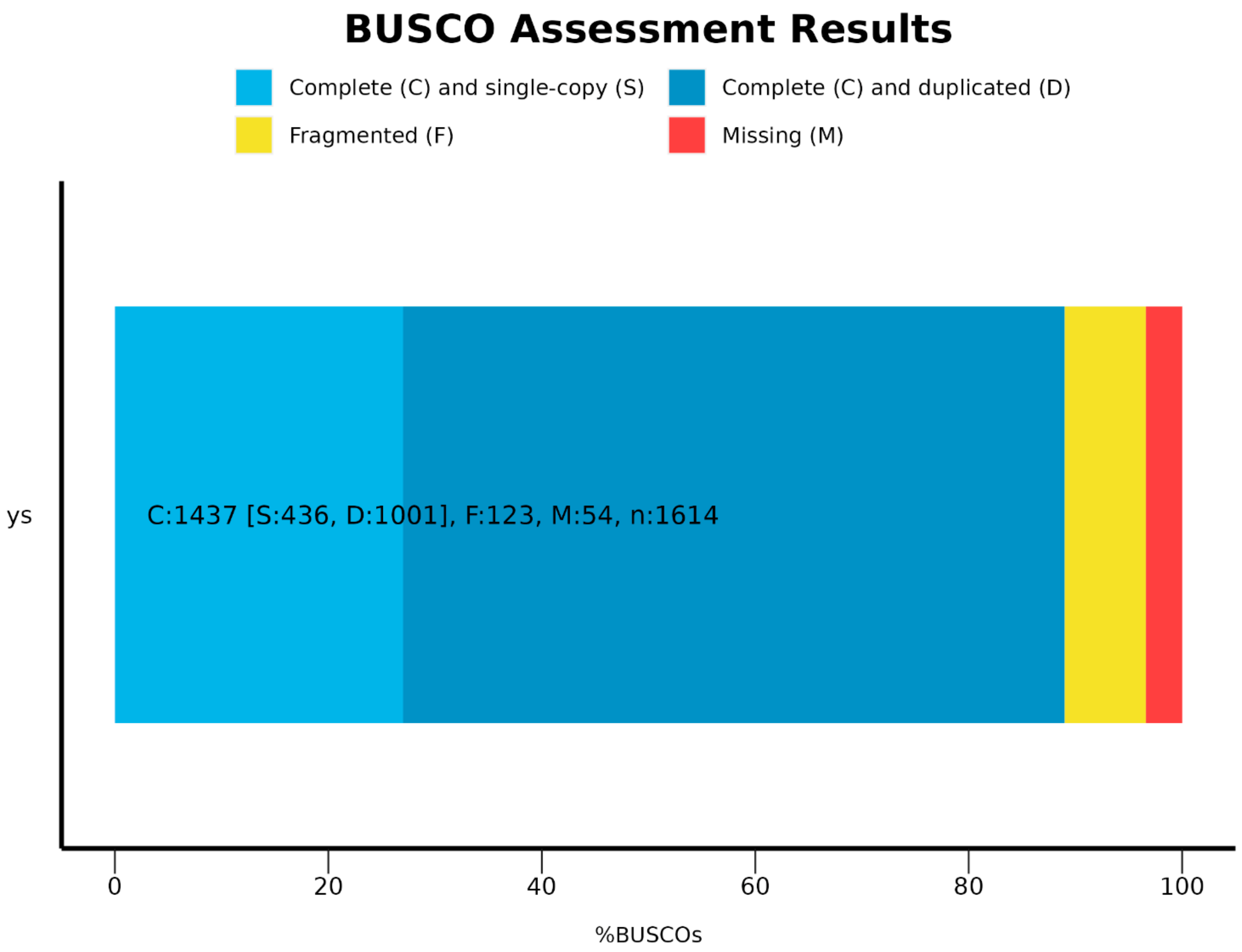

Figure S2. Differentially expressed genes (DEGs) among different color types.
